# Supplementary material for: MetaQTL: a package of new computational methods for the meta-analysis of QTL mapping experiments
Source: BMC Bioinformatics. 2007 Feb 8;8:49. doi: 10.1186/1471-2105-8-49 (PMC1808479; doi:10.1186/1471-2105-8-49)
Supplement: Additional File 7 — MetaQTL Package : jar file and tutorial. This Zip archive contains both the MetaQTL JAR file and the files of the tutorial. [file 1471-2105-8-49-S7.zip › org.inra.metaqtl/doc/org/thalia/bio/entity/factory/class-use/BioEntityFactory.html]

Uses of Class org.thalia.bio.entity.factory.BioEntityFactory


|  |  |  |
| --- | --- | --- |
| **Overview** \| **Package** \| **Class** \| **Use** \| **Tree** \| **Deprecated** \| **Index** \| **Help** | |  |
| PREV   NEXT | **FRAMES**    **NO FRAMES**     **All Classes** |  |


---


## **Uses of Class org.thalia.bio.entity.factory.BioEntityFactory**

| Packages that use BioEntityFactory | |
| --- | --- |
| **org.thalia.bio.entity.factory** |  |

Uses of BioEntityFactory in org.thalia.bio.entity.factory

| Subclasses of BioEntityFactory in org.thalia.bio.entity.factory | |
| --- | --- |
| `class` | `MapChartFactory`             General comments must be added here. |
| `class` | `MapMakerFactory` |
| `class` | `MapRawFactory` |
| `class` | `MapTabFactory` |
| `class` | `OntologyTabFactory`             A factory to deal with `Ontology`. |
| `class` | `XmlBioEntitiesFactory`             Class Description Here |
| `class` | `XmlBioEntityFactory`             Class Description Here |
| `class` | `XmlGeneticMapFactory`             Class Description Here |
| `class` | `XmlOntologyFactory` |

---


|  |  |  |
| --- | --- | --- |
| **Overview** \| **Package** \| **Class** \| **Use** \| **Tree** \| **Deprecated** \| **Index** \| **Help** | |  |
| PREV   NEXT | **FRAMES**    **NO FRAMES**     **All Classes** |  |


---
